# Predictors for readmission risk in schizophrenia: insights from a Saudi Arabian cohort

**DOI:** 10.3389/fpsyt.2025.1593653

**Published:** 2025-09-16

**Authors:** Mahmoud A. Alharthi, Rajaa M. Al-Raddadi, Sulhi A. Alfakeh

**Affiliations:** ^1^ Population Health Management, Jeddah First Health Cluster, Jeddah, Saudi Arabia; ^2^ Department of Community Medicine, Faculty of Medicine, King Abdulaziz University, Jeddah, Saudi Arabia; ^3^ Psychiatry Division, Department of Internal Medicine, Faculty of Medicine, King Abdulaziz University, Jeddah, Saudi Arabia

**Keywords:** patient readmission, schizophrenia, mental health, length of stay, retrospective studies, psychiatric department, Saudi Arabia

## Abstract

**Background:**

The readmission of individuals with schizophrenia to inpatient care can pose significant challenges and disturb the lives of both patients and their families, as well as the functioning of mental health care systems. Despite the growing prevalence of schizophrenia, there is limited research focused on the predictors of readmission within the Saudi Arabian context.

**Objectives:**

The primary objective of this research is to assess the readmission rate for patients who have been diagnosed with schizophrenia, as well as to determine the associations between socio-demographic factors and the risk of readmission for these patients.

**Methodology:**

In a retrospective cohort study, 145 individuals who were admitted to the Eradah and Mental Health complex in Jeddah since July 1st to December 31st in 2018 were recruited. Data on socio-demographic characteristics, medical history, medication adherence, and follow-up care were analyzed to determine their association with one-year readmission rates.

**Results:**

Findings revealed that male participants comprise 84.8% of the sample size. Moreover, 5.5% of the patients were employed, with the larger proportion being unemployed. The overall one-year readmission rate was 36.6%. The Key predictor of readmission included the number of previous admissions, length of hospital stay and frequency of outpatient follow-up visits. After controlling to other variables in a multivariate model, the length of stay was not statistically significant, despite appearing to be related to readmission in the unadjusted analysis. The probability of readmission was significantly increased by inadequate follow-up, illustrating the vital role of continuous post- discharge care.

**Conclusions:**

According to recent research, improving the transfer from inpatient psychiatric care to outpatient care may lower the risk of readmissions and prevent future hospitalization. The results emphasize the importance of specific treatments in minimizing readmission rates among schizophrenia patients in Saudi Arabia.

## Graphical Abstract

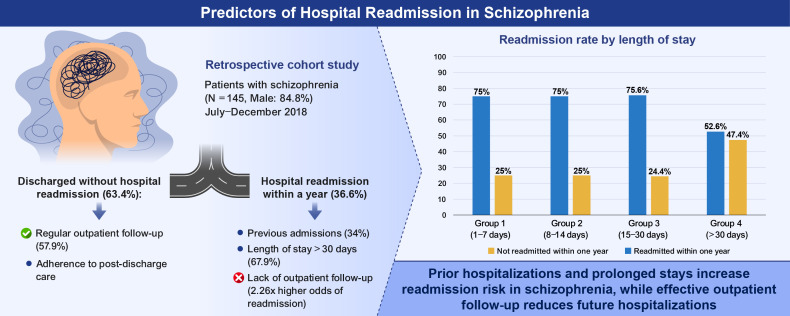

## 1 Introduction

Schizophrenia is a severe and chronic psychiatric disorder characterized by significant abnormalities in perception, emotions, language, self-identity, behavior and thought processes. Two symptoms of schizophrenia are phantasmagoria, which are auditory or visual experiences without external stimuli and delusions, which are enduring misconceptions that are unaffected by contradicting information. Also, patients may covey feelings of apathy outside influence or external control. Many studies have found that this disease is associated with a variety of motor symptoms (1). Patients with altered behavioral patterns generally have delayed motor initiation, slower psychomotor responses and irregular involuntary motions, which ultimately results in a loss of life purposes (2).

According to the 2011World Report on Disability, schizophrenia ranks as the 15th most disabling condition (3). This study, which was co-authored by the WHO demonstrates a clear connection between mental illness and its impact on people’s social and economic well-being. People who suffer from mental illness are not the only ones affected their family are also impacted. As a result, schizophrenia can be characterized detrimental effect on both the healthcare system and society’s overall health.

Schizophrenia is a severe national health issue that can have a significant negative impact on people’s lives, society and economy. Establishing continuing care plans is essential in this case to prevent relapses following intensive care (4).

The number of cases with schizophrenia increased from 13.1 million in 1990 to 20.09 million in 2016 (5). Because of its prevalence it may be estimated that 1.5 out of every 10,000 people have this condition on average, and that its incidence is higher than that of other psychomotor disabilities (6). According to 2024 by Alangari et al., based on Saudi Arabian cohort females are 2.2% more likely than men to experience mental morbidities. Between March 2022 to March 2023, 46 primary care facilities in Riyadh, Saudi Arabia, provided the data used in this investigation.

About 13% of patients receiving treatment at psychiatric outpatient clinics in Saudi Arabia have schizophrenia in one form or another. 13,363 new and frequent cases were hospitalized to psychiatric inpatient departments while 145,625 new and frequent cases visited psychiatric outpatient clinics, according to this Saudi MOH delusional disorders, schizotypal disorders and schizophrenia were identified in each of these individuals (7). these figures demonstrates the serious threat this illness poses to Saudi citizens public health.

The WHO measures the comprehensive disease trammel using DALYs, this includes the total number of years of life lost owing to premature death and the years spent disabled due to disease or disability (8). This burden of disease is well recognized worldwide according to 1993 GBD report.

South Korea had an approximately 1.13 million cases of schizophrenia in 2017, with a burden of 12.66 DALYs per 100,000 people, according to DALY metric. In comparison the global average for the same year was 195.27 DALYs per 100,000 people (9, 10). Specifically, in Saudi Arabia was expected to have 124.89 DALYs from schizophrenia per 100,000 individuals in 2017, 36% lower than the global average for that year (11).

Schizophrenia put a significant strain on families because to the high rate of readmissions. There is a favorable correlation between readmissions and the diagnosis of schizophrenia (12) and this relationship has been extensively studied throughout the years.

Dionisie et al.’s study which looked at the medical records of 8,705 patients with schizophrenia over a 10 year period, produced notable findings. their study found a positive correlation between the, positive correlation between the number of prior admissions and risk of readmission. Specifically, patients who were readmitted after being released from hospital had a significantly increased chances of being readmitted again between their second and fifteenth readmissions, Conversely, those who never experienced readmission had a very low chance of doing so in the future (13).

Further research has connected relapses to adverse outcomes, including decreased psychosocial and vocational functioning (14). Patients with schizophrenia are prone to relapses even after completing course, and many readmissions are expected following their initial psychotic episodes (15).

Readmission can have detrimental effects on patients and their loved ones such as higher cost for mental health care and distressing events. In the literature, this condition has been categorized as, “relapse and recidivism,” by a number of researchers emphasizing the costly nature of repeated inpatient hospitalizations episodes (16, 17).

For a medical industry, the readmission rate is a vital global indicator of the prevalence of “relapse and recidivism” (18). This indicator serves as a qualitative evaluation tool and draws the interest of healthcare officials (19). Furthermore, readmission should be interpreted as a symptom that a patient’s condition has gotten worse or that their illness has returned (20).

A substantial portion of peoples with schizophrenia are readmitted after being discharged, according a 2011 Organization for Economic Cooperation and Development (OECD) study that was carried out in 15 countries. This study found that after 30 days, the overall unplanned readmission rate among discharged patients with schizophrenia was 13% (16). Another study determined that the 30-day readmission rate to psychiatric institutions in six European countries was 10.4% (20, 21). In Korea, 46.8% of patients with schizotypal, schizophrenia and delusional disorders were expected to be readmitted within 180 days in 2017 22). A similar investigation conducted in Oman revealed that 39% of patients were readmitted within 1-year after their initial hospitalization (22). A study conducted in Saudi Arabia revealed a readmission rate 83.3% among patient with schizophrenia treated in four central mental facilities affiliated with the MOH, indicating significant public health concern for this population (23).

Research has shown that there are other factors associated with psychiatric readmissions besides the severity of mental illness (24). One factor under investigation is the duration of inpatient stay, which has been associated with a higher readmission rate (25, 26). However, different European countries have different effects from length of residence. for example, longer stays are linked to lower rates of psychiatric rehabilitation in Finland and Norway, but the converse is true in Romania.

This study aimed to identify the variables that affect the readmission rates of patients with schizophrenia following their discharge from an inpatient facility in Saudi Arabia. By identifying these predictors, we may improve healthcare programs and ultimately improve the lives of local people with schizophrenia.

## 2 Methods

This retrospective cohort study was designed and conducted at the Eradah and Mental Health Complex in Jeddah, Saudi Arabia, from July 1 and December 31, 2018, to examine medical data for for patients who were discharged within a 1-year. The Eradah and Mental Health Complex is a 125-bed mental health facility that provides a variety of psychological treatments, including as inpatient treatment, home care, emergency care and psychiatric outpatient.

### 2.1 Research plan

The study design was retrospective and was based on a detailed examination of the medical records of people who had been diagnosed with schizophrenia. The analysis was performed in accordance with the Strengthening the Reporting of Observational Studies in Epidemiology (STROBE) guidelines to generate data regarding schizophrenia that could be used broadly in the Saudi Arabian context (27).

### 2.2 Patient selection criteria

The study cohort consist of patients assessed with schizophrenia who were later hospitalized to the Eradah and Mental Health Complex. The date of initial admission is designated as the index and first admission. The investigative period spanned from July 1 to December 31, 2018.

Post-discharge, the enrolled patients were observed for duration of 1-year to identify any incidences of readmission. The study for any designated patient concluded upon the completion of 1-year from their first admission. For example, for a schizophrenia patient admitted on August 1, 2018, observations would conclude on July 30, 2019.

The inclusion strategy for this research was as follows: (i) patients diagnosed with schizophrenia based on the DSM-IV (27). DSM-IV delineates differential levels of criteria to be assessed by researchers in relation to mental disorders; (ii) patients with 18 years or above; (iii) those who were clinically stable; and (iv) those whose hospitalization duration aligned with the study timeframe.

Patients with significant physical comorbidities such as cardiovascular diseases, uncontrolled diabetes, neurological disorders (e.g. epilepsy) or any chronic illnesses requiring hospitalization were excluded from the study, along with those having legal issues or who had died.

### 2.3 Data collection and instrumentation

Socio-demographic and health data were primarily retained from the therapeutical records of patients from the aforementioned facility. The collected data encompassed patient data, including educational level, age, living situation, sex and marital status. The living situation was further evaluated based on patients’ employment status and income, as well as whether they received social security benefits and demonstrated signs of inadequate social support. Furthermore, data regarding admissions and readmissions were recorded, specifying whether they were voluntary or involuntary with regard to patient consent.

In addition to the aforementioned data, critical information relevant with psychiatric disorders was taken into account, including incidences of self-harm, aggressive behaviors, substance abuse, adherence to medication, comorbidities, with the application of injectable, long-acting antipsychotics. All these parameters were considered concerning patient discharge.

Data collection occurred during a defined period, from July 1 to December 31, 2018. Moreover, the data were initially captured in printed format and subsequently input into the Epi Info program version 7, evolved by the Centers for Disease Control and Prevention.

Some variables had incomplete data; participants with missing values for specific variables were excluded listwise from that analysis, resulting in slight variations in sample size across some tables.

Readmission was defined as any unplanned inpatient psychiatric hospitalization occurring within 1-year of discharge from the index admission planned readmissions, such as scheduled admissions for treatment review or medication adjustment were excluded.

### 2.4 Primary study outcome

Assessing a year of psychiatric readmission rates for individuals with schizophrenia after their first discharge was the main goal of this study.

### 2.5 Statistical analysis

Descriptive statistics were used to summarize socio-demographic and clinical characteristics. Means and SD were reported for continuous variables and frequencies and percentages were used for categorical variables. Bivariate associations between categorical variables and readmission were assessed using Fisher’s exact test or Chi-Square as pertinent.

Variables that showed a statistically significant association with readmission in the bivariate analysis (p< 0.05) were considered for inclusion in the multiple logistic regression models. The final model adjusted for number of previous admissions, length of inpatient stay and outpatient follow-up status. These variables were selected based on their significance in the bivariate analysis and clinical relevance supported by prior literature.

The quantity of variables used in the regression model was carefully constrained according to the small sample size n=145 in order to lower the possibility of model overfitting. Statistical significance was defined as a P=value of less than 0.05. Version 28 of IBM SPSS statistics was used for all procedures.

To evaluate the discriminative ability of the logistic regression model, a receiver operating characteristic (ROC) curve was marked using predicted probabilities. AUC was computed, with a value >0.7 considered acceptable for model discrimination.

### 2.6 Ethical approval

All information gathered for this study was obtained through legal and cooperative processes compliant with MOH guidelines. The ethical approval for this study was granted on April 27, 2020, by the IRB of the MOH (registration number: *H-02-J-002*). Written informed consent was obtained from all patients or their legal guardians. The study adhered to GCP guidelines. No hard copies of participant data were retained, and access to the data was restricted to the investigator. The collected data were securely saved on a password-protected drive within the individual’s computer, which also employs internet security and antivirus software, thus ensuring the privacy and protection of patient information.

## 3 Outcomes

### 3.1 Socio-demographic characteristics

A sum of 145 adult patients diagnosed by schizophrenia who were hospitalized into inpatient wards of the Eradah and Mental Health Complex during the period from July 1 to December 31, 2018, were included in the study. As demonstrated in **Table 1**, the majority of the participants were male, accounting for 84.8% of the study population. The mean age of all patients was 34.2 years (± 10.2), ranging from 18 to 64 years. Notably, a significant proportion of patients (37.2%) fell within the age range of 18–29 years.

**Table 1:** Baseline Characteristics of the study population.

Variable	n	%
Sex: Male	123	84.8
Sex: Female	22	15.2
Age: 18–29	54	37.2
Age: 30–39	50	34.5
Age: ≥40	41	28.3
Education: Illiterate	8	5.5
Education: < High school	62	42.8
Education: High school	54	37.2
Education: Graduate	21	14.5
Marital: Married	20	13.8
Marital: Divorced/Widowed	24	16.5
Marital: Single	101	69.7
Living: Alone	20	13.9
Living: With family	122	84.7
Living: Institutionalized	2	1.4
Employment: Employed	8	5.5
Employment: Retired	11	7.6
Employment: Unemployed	126	86.9
Income(SR): <3000	117	80.7
Income: 3000– less than5000	9	6.2
Income: ≥5000	9	6.2
Social Security: No	60	43.5
Social Security: Yes	78	56.5
Illness duration <5 yrs	38	26.8
Illness 5–10 yrs	54	38
Illness >10 yrs	50	35.2
Comorbidity: No	96	66.2
Comorbidity: Yes	49	33.8
Medication compliance: No	124	87.3
Medication compliance: Yes	18	12.7
Long-acting antipsychotic: No	19	13.1
Long-acting antipsychotic: Yes	126	86.9
Electroconvulsive therapy: No	112	77.2
Electroconvulsive therapy: Yes	33	22.8
Substance abuse: No	89	61.4
Substance abuse: Yes	56	38.6
Aggression (risk to others): No	92	63.4
Aggression: Yes	53	36.6
Self-harm risk: No	134	92.4
Self-harm risk: Yes	11	7.6
Poor social support: No	114	78.6
Poor social support: Yes	31	21.4
No previous admission	36	25
One admission	36	25
Two admissions	17	11.8
Three admissions	25	17.4
More than three admissions	30	20.8
Compulsory admission: No	86	59.3
Compulsory admission: Yes	59	40.7
Discharge type: Planned	137	94.5
Discharge type: Unplanned	8	5.5
Readmission: No	92	63.4
Readmission: Yes	53	36.6
Follow-up: No	61	42.1
Follow-up: Yes	84	57.9
Outpatient visits: No visit	61	42.1
Outpatient visits: One	34	23.4
Outpatient visits: Two	29	20
Outpatient visits: Three	11	7.6
Outpatient visits: >Three	10	6.9

SR, Saudi Riyal; percentage may not total 100 due to rounding.

Regarding educational attainment, the majority of participants (48.3%) had not completed high school. Additionally, a vast majority (86.2%) were unmarried. Moreover, 84.7% of patients resided with their families. Data regarding accommodation indicated that 27% of patients lived outside Jeddah, while only 5.5% were employed. A considerable percentage (80.7%) reported earning less than 3,000 SR per month, with a mean income of 1501.1 SR (SD 2192.5 SR), and 43.5% of patients did not receive social security salaries.

### 3.2 Clinical characteristics

In the study population, the time span of sickness was 9.7 years (SD 7.14 years), ranging from 1 to 38 years. Approximately 73.2% of patients had been ill for 5 years or more, as represented in **Table 1**. Furthermore, a comorbidity was absent in 66.2% of the patients. Concerning medication compliance, 87.3% of patients exhibited non-compliance. All participants received oral antipsychotic treatments, and 86.8% were administered long-acting injectable antipsychotics, as indicated in **Table 1**. Notably, 77.2% of patients had not undergone electroconvulsive therapy sessions, and 61.4% of patients had no recorded history of substance abuse. Additionally, 63.4% of patients posed no risk to others, while a significant majority (92.4%) presented no risk to themselves. Lastly, 78.6% of patients did not show evidence of inadequate social support in their medical records.

**Table 1** indicates that approximately half of the study participants (50%) had a history of multiple hospital admissions. Moreover, the largest proportion of patients (59.3%) were involuntarily admitted under compulsory care. In terms of discharge planning, a substantial majority (94.5%) were discharged with a structured discharge plan. However, a notable third of patients admitted during the study period experienced readmission within a year of their initial discharge, resulting in an overall readmission rate of 36.6%.

The proportion of patients receiving follow-up care post-discharge was found to be 57.9% (**Table 1**). Additionally, it was found that 14.5% of these patients made three or more visits to outpatient clinics within 1-year of discharge from inpatient wards.

### 3.3 Risk factors of readmission

As demonstrated in **Table 2**, no statistically significant variations were observed among admitted patients concerning factors such as sex, age, education, marital status, and living conditions. Similarly, no notable disparities in employment status, income, and social security benefits were identified.

**Table 2:** Bivariate analysis of the association between socio-demographic variables and readmissions among the study population (n=145).

Variable		Readmitted (n, %)		Not Readmitted (n, %)		P-value
Sex	Male	76	61.8	47	38.2	0.32 ^a^
	Female	16	72.7	6	27.3	
Age (in years)	18 – 29	36	66.7	18	33.3	0.61 ^a^
	30 – 39	29	58	21	42	
	≥40	27	65.9	14	34.1	
Education level	Illiterate	3	37.5	5	62.5	0.16 ^b^
	Less than high school	38	61.3	24	38.7	
	High school	34	63	20	37	
	Graduate	17	81	4	19	
Marital status	Married	16	80	4	20	0.24 ^b^
	Divorced and widowed	15	62.5	9	37.5	
	Single	61	60.4	40	39.6	
Living condition (n=144)	Alone	14	70	6	30	0.82 b
	With family	76	62.3	46	37.7	
	Institutionalized	1	50	1	50	
Employment status	Employed	7	87	1	12.5	0.32 ^b^
	Retired	6	54.5	5	45.4	
	Unemployed	79	62.7	47	37.3	
Income (Saudi Riyal) (n=135)	< 3000	71	60.7	46	39.9	0.73 ^b^
	3000 – < 5000	6	66.7	3	33.3	
	≥5000	7	77.8	2	22.2	
Receiving social security salary (n=138)	No	39	65	21	35	0.67 ^a^
	Yes	48	61.5	30	38.5	

P-values calculated using Chi-square test unless otherwise specified. Fisher’s exact test was used when Chi-square assumptions were not met. Data compares patients readmitted within 1-year vs those not readmitted.

^a^Chi-square test; ^b^Fisher’s exact test (used when Chi-square assumptions were not met or expected cell counts were small).

According to the clinical characteristics presented in **Table 3**, the duration of illness, presence of comorbidity (OR = 1.45, 95% CI:0.75-2.78,p=0.26), and medication compliance (OR = 0.61,95% CI:0.21-1. 81,p=0.37) showed no statistically significant association with readmission. Furthermore, no significant association was noted between long-acting antipsychotics use and readmission (OR = 0.98, 95% CI:0.41-2.34,p=0.97). Electroconvulsive therapy showed no statistically significant association with readmission (OR = 1.18, 95% CI:0.51-2.56,p=0.97). Substance abuse (OR = 1.84, 95% CI:0.90-3.76,p=0.10)., risk to self (OR = 2.25, 95% CI:0.68-7.74,p=0.21) and inadequate social support (OR = 1.31, 95% CI:0.61-2.80,p=0.48) revealed no statistically significant associations with readmission.

**Table 3:** Bivariate analysis of the association between clinical variables with readmissions among study population (n=145).

Variable		Readmitted (n, %)		Not Readmitted (n, %)		P-value
Illness duration (in years) (n=142)	< 5 years	27	71.1	11	28.9	0.43 ^a^
	5–10 years	33	61.1	21	38.9	
	> 10 years	29	58	21	42	
Presence of comorbidity	No	64	66.7	32	33.3	0.26 ^a^
	Yes	28	57.1	21	42.9	
Compliance to medication (n=142)	No	76	61.3	48	38.7	0.37 ^a^
	Yes	13	72.2	5	27.8	
Receiving long-acting injectable antipsychotic	No	12	63.2	7	36.8	0.97 ^a^
	Yes	80	63.5	46	36.5	
Electroconvulsive therapy	No	72	64.3	40	35.7	0.70 ^a^
	Yes	20	60.6	13	39.4	
Substance abuse	No	61	68.5	28	31.5	0.10 ^a^
	Yes	31	55.4	25	45.6	
Aggression (Risk to others)	No	59	64.1	33	35.9	0.82 ^a^
	Yes	33	62.3	20	37.7	
Risk of self-harm	No	87	64.9	47	35.1	0.21 ^a^
	Yes	5	45.5	6	54.5	
Poor social support	No	74	64.9	40	35.1	0.48 ^a^
	Yes	18	58.1	13	41.9	

P-values calculated using Chi-square test unless otherwise specified. Fisher’s exact test was used when Chi-square assumptions were not met. Data compares patients readmitted within 1-year vs those not readmitted.

^a^Chi-square test.

An examination of the counting of prior admissions revealed significant association with readmissions, as illustrated in **Table 4**, with a p-value of 0.04. Approximately 34% of the readmitted patients had experienced more than three prior admissions. However, no significant associations were found between readmission and compulsory first admissions; and that between readmissions and the type of discharge (whether planned or unplanned), as indicated in **Table 4**. Notably, there was no significant association was found in the middle of discharge type from first admission, as 62.5% of patients that were discharged unplanned, subsequently experienced readmission.

**Table 4:** Bivariate analysis of the association between admissions variables and readmissions (n=145).

Variable		Readmitted (n, %)		Not Readmitted (n, %)		P-value
Previous admissions (n=144)	No previous admission	25	69.4	11	30.6	0.04* ^a^
	One admission	27	75	9	25	
	Two admissions	11	64.7	6	35.3	
	Three admissions	16	64	9	36	
	More than three admissions	12	40	18	60	
Compulsory first admission	No	57	66.3	29	33.7	0.39 ^a^
	Yes	35	59.3	24	40.7	
Type of the first discharge	Planned	89	65	48	35	0.14 ^b^
	Unplanned	3	37.5	5	62.5	
Length of stay groups	Group one (1–7 days)	3	75	1	25	0.04* ^b^
	Group two (8–14 days)	18	75	6	25	
	Group three (15–30 days)	31	75.6	10	24.4	
	Group four)>30 days)	40	52.6	36	47.4	

P-values calculated using Chi-square test unless otherwise specified. Fisher’s exact test was used when Chi-square assumptions were not met. Data compares patients readmitted within1-year vs those not readmitted.

Bold values indicate statistical significance at p < 0.05.

^a^Chi-square test; ^b^Fisher’s exact test (used when Chi-square assumptions were not met or expected cell counts were small).

**Figure 1:**
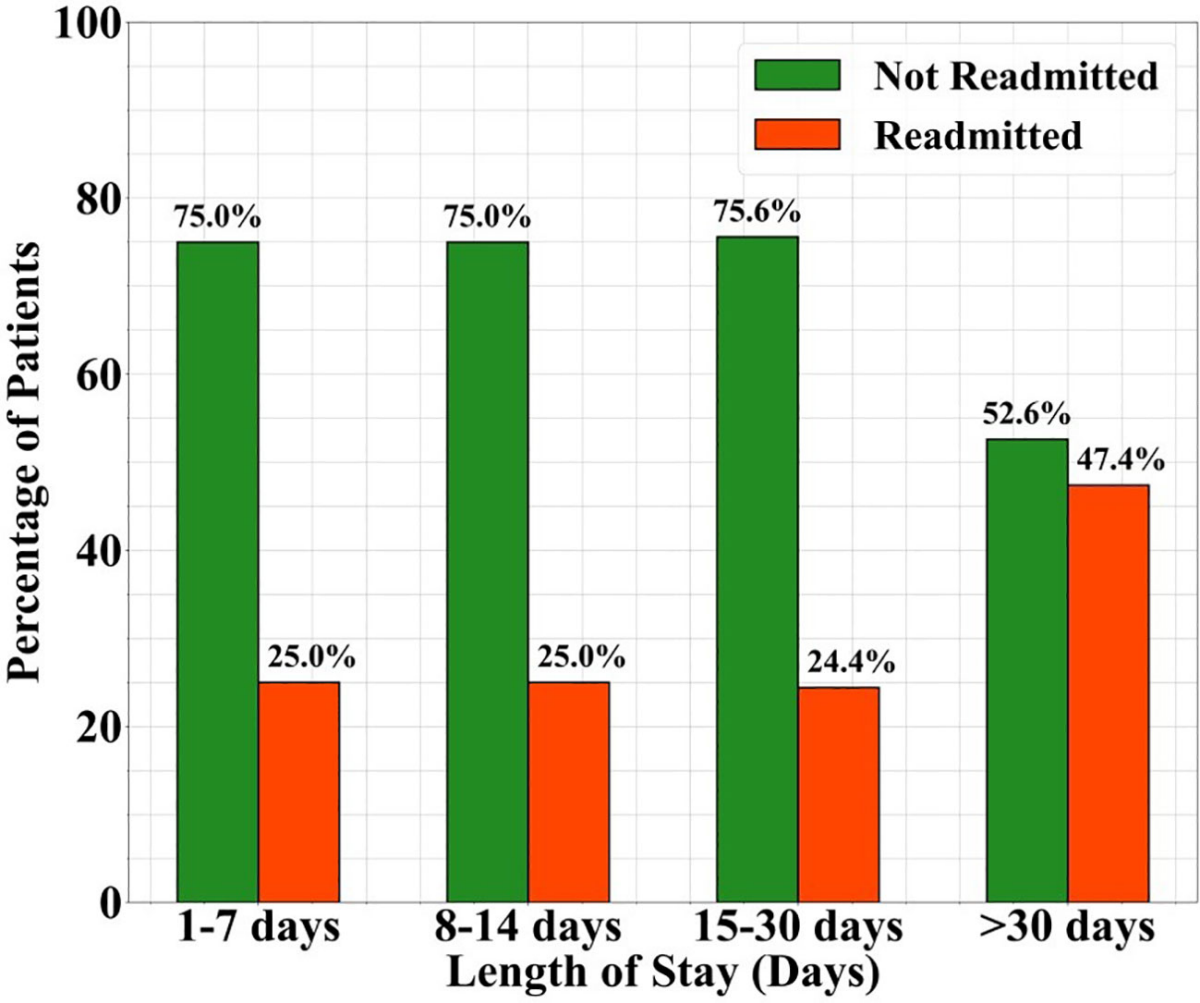
Readmission rates by length of stay.

**Table 4** indicates that the length of stay variable was significantly associated with readmissions (p=0.04). **Figure 1** shows the proportion of patients readmitted within 1-year following discharge categorized by the duration of their initial hospital stay. Patients with longer hospital stay (>30 days) sowed a higher readmission rate (47.4%) compared to those with shorter stays.

The results in **Table 5** indicate that the “outpatient care follow-up” variable was significantly associated with readmission (p<0.001). Furthermore, a significant relationship emerged between the number of outpatient care visits and readmission outcomes (p=0.02).

**Table 5:** Bivariate analysis of the association between outpatient follow-up variables and readmission rates among the study population (n=145).

Variable		Readmitted (n, %)		Not Readmitted (n, %)		P-value
Follow-up (outpatient care)	No	29	47.5	32	52.5	< 0.001* ^a^
	Yes	63	75	21	25	
Number of visits to outpatient clinics within 1-year	No visit	29	47.5	32	52.5	0.02* ^b^
	One visit	25	73.5	9	26.5	
	Two visits	21	72.4	8	27.6	
	Three visits	9	81.8	2	18.2	
	More than three visits	8	80	2	20	

P-values calculated using Chi-square or Fisher’s exact test, as appropriate. Data compares patients readmitted within 1-year vs. those not readmitted.

Bold values indicate statistical significance at p < 0.05.

^a^Chi-square test; ^b^Fisher’s exact test (used when Chi-square assumptions were not met or expected cell counts were small).

### 3.4 Logistic regression

The unadjusted odds ratios derived from the simple logistic regression analyses are presented in **Table 6**. These odds ratios assess the association between independent variables (risk factors for readmission) and the dependent variable (readmission) without controlling for other variables. Risk factors such as previous admissions, length of stay, and follow-up (outpatient care) emerged as independent covariates demonstrating significant correlations with readmission when subjected to bivariate analysis.

**Table 6:** Crude odds ratio from logistic regression analysis identifying the association between risk factors and readmission.

Predictor variable	Crude OR	95% CI		P-value
		Lower limit	Upper limit	
Previous admissions				
No previous admission (ref)				
One admission	0.758	0.269	2.133	0.599
Two admissions	1.240	0.365	4.206	0.730
Three admissions	1.278	0.433	3.770	0.656
More than three admissions	3.409	1.232	9.436	0.018*
Length of stay				
Group one (1–7 days) (ref)				
Group two (8–14 days)	1.000	0.087	11.525	1.000
Group three (15–30 days)	0.968	0.090	10.381	0.978
Group four)>30 days)	2.700	0.269	27.134	0.399
Follow-up to outpatient care				
Yes (ref)				
No	**3.098**	1.534	6.257	0.002*

Ref, reference category; *statistically significant.

Bold values indicate statistical significance at p < 0.05.

The results of multiple logistic regression produced adjusted odds ratios, as outlined in **Table 7**. These adjusted ratios assess the relationship between the independent variables (namely, susceptibility factors for readmission) and the dependent variable (readmission), after taking into account other variables within the model. **Table 7** presents multiple logistic regression analysis aimed at investigating the associations between prior admissions, length of stay, and follow-up (outpatient care) on the one hand, and readmission on the other. For the variable concerning outpatient care follow-up, the “yes” category was adopted as the reference group. The analysis indicated a significant association between readmission rates and follow up (outpatient care) compared to no follow-up. Notably, the odds of readmission were 2.266 times higher among those who failed to attend outpatient follow-ups compared to those who did follow up, after adjustments for other variables within the model. This logistic regression analysis emphasizes a significant correlation between outpatient care follow-up and readmission risk.

**Table 7:** Adjusted odds ratio from logistic regression analysis identifying the association between risk factors and readmission.

Predictor variable	AOR	CI (95%)		P–value
		Lower limit	Upper limit	
Previous admissions				
No previous admission (ref)				
One admission	0.613	0.203	1.850	0.385
Two admissions	1.012	0.279	3.677	0.985
Three admissions	0.853	0.266	2.743	0.790
More than three admissions	2.082	0.686	6.315	0.195
Length of stay				
Group one (1–7 days) (ref)				
Group two (8–14 days)	0.973	0.077	12.341	0.983
Group three (15–30 days)	0.887	0.076	10.417	0.924
Group four)>30 days)	2.177	0.196	24.164	0.527
Follow-up (outpatient care)				
Yes (ref)				
No	**2.266**	1.047	4.903	0.038*

Ref, reference category; OR, Odd Ratio; AOR, Adjusted Odd Ratio; CI, Confidence Interval P<0.05 considered statistically significant. Bold values indicate significance. The logistic regression model was adjusted for number of previous admissions, length of stay, and outpatient follow-up status.

Bold values indicate statistical significance at p < 0.05.

Despite being linked to readmission in the bivariate approach, the period of care and prior hospitalizations were no longer statistically essential in the adjusted model. For instance, readmission following equalization was not substantially correlated with length of stay greater than 30 days (AOR = 2.18, 95% CI: 0.20-24.16,P = 0.527), nor was having more than three previous admissions (AOR = 2.08, 95% CI: 0.69-6.31, P = 0.195).

### 3.5 ROC curve Analysis

**Figure 2:**
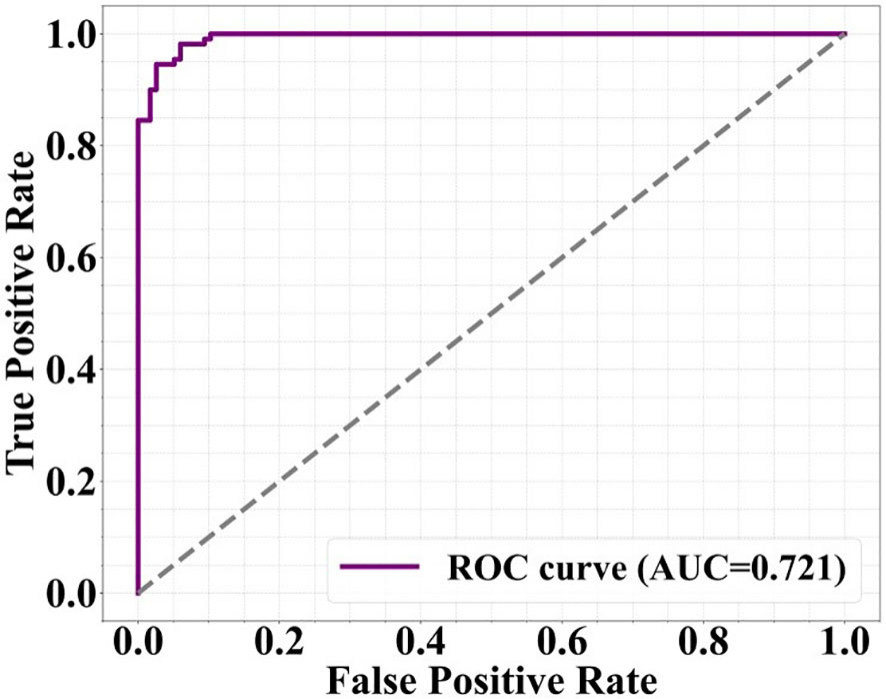
ROC curve for the logistic regression model predicting readmission. The area under the curve was 0.721.

The ROC curve analysis (**Figure 2**) was conducted to determine the predictive performance of the logistic regression model. The resulting area under the curve (AUC) was 0.721, indicating an acceptable level of discrimination between patients who were readmitted and those who were not. This suggests that the model had a fair ability to correctly classify readmission outcomes.

## 4 Discussion

The present investigation focused to elucidate the predictors of readmission risk out of 145 patients diagnosed with schizophrenia within the specific context of the Saudi Arabian healthcare setting and to detect socio-demographic and clinical factors associated with one-year readmission rates. The findings revealed an overall readmission rate of 36.6%, with significant predictors identified, including the history of previous admissions, the length of inpatient stay, and the frequency of outpatient follow-up visits. Notably, inadequate follow-up was found to significantly increase the likelihood of readmission, underscoring the critical role of continuous care in improving patient health impact.

The findings of this study align with previous research. The current work illustrates socio-demographic parameters, drawing initial comparisons with the study by Parentela et al., which was situated in a Saudi Arabian context (23). The current study identified that the largest proportion of readmitted patients were unmarried (92.5%), and 73.6% were aged <40 years, with a mean age of 34.2 years. Furthermore, 90.2% of patients reported an income below 3,000 SR. Parentela et al.’s study involved a cohort of 156 participants, yielding similar demographic outcomes (23); however, the restriction to exclusively male subjects prevented direct sex comparisons.

Notably, the findings of this study also echo those of Yang et al., who examined the Taiwanese population (28). Yang et al. found a predominance of younger individuals within the readmitted group, with a mean age of 35.9 years, and similarly identified an increased prevalence of unmarried patients and male sex, consistent with the current results. It is crucial to recognize that while demographic variables in this study are based on a Saudi Arabian population, Hung’s analysis centered on a Taiwanese cohort. Moreover, attempts to contextualize findings within a global framework indicate overlap with research conducted by Gonçalves-Pinho et al. (29), which reported that male patients experienced higher frequencies of readmissions, corroborating the current study’s outcomes.

In contrast to the findings of de Jong et al. (30). Female patients in this study appeared less likely to be readmitted. However, this result should be interpreted cautiously, as the female subgroup in our sample was relatively small (15.2%) and may not adequately represent broader gender-related readmission trends. The observed difference could be influenced by sample selection bias or institutional factors affecting admission patterns. From an educational perspective, similarities were noted regarding the educational characteristics of the readmission group with the observations of Levine and Lyngstad et al. (31). Patients who did not finish school represented 54.7% of our study population. On the contrary, no significant differences were observed concerning essential demographic factors between the readmission and non-readmission groups, corroborating findings from Kessler and Lev-Ran (32). Similarly, a study conducted by Martinho et al. found no differential values when analyzing demographic factors against readmission rates (33).

In terms of clinical factors, this study found no significant correlation concerning the type of first hospital admission (voluntary vs. involuntary) and evidence of poor social support among readmitted patients. Our study found that the patients who were admitted under compulsory care or with poor social support were less frequent into readmitted group; however, there was no statistical significance was found. These findings differ from some global studies where similar analyses yielded dissimilar results. Given the variability inherent in cohort and cross-sectional studies, the presence of dissimilarity in the literature warrants acknowledgment.

Studies by Schmitz-Buhl et al. and Virtanen et al. indicated no correlation between high 1-year readmission risk and involuntary admissions (34, 35). Conversely, research by de Girolamoet al. and Han et al. identified that physically aggressive patients faced higher readmission rates compared to those exhibiting no aggressive behaviors, with significance reported by the researchers (36, 37). In contrast, this current work found no such significant relationship between aggressive behaviors and readmission rates.

Additionally, hospital admissions prior to the first admission date were shown to bear no significant association with readmission rates, contradicting the findings presented by Hou et al. (38). Moreover, this investigation yielded no evidence of self-harm-related readmission risk among patients discharged from psychiatric inpatient care, differing from the results reported by Lee et al. (39).

These divergences from global finding may be attributed to differences in healthcare infrastructure and cultural practices in Saudi Arabia. For instance, the reliance on family-based car, limited access to long-term community psychiatric support, and stigma surrounding mental illness may influence hospitalization and follow-up patterns differently compared to western settings. Additionally, variation in discharge planning protocols and outpatient service integration could contribute to discrepancies in readmission outcomes.

Furthermore, the analysis of this study revealed no significant impact of comorbidity or substance abuse on readmission rates, opposing the findings from Cook et al. (40). Despite previous research linking poor compliance to treatment regimens with relapse and readmission risk, the results of this study did not yield statistically significant associations with these factors (41, 42).

Despite medication adherence will not show a statistically significant association with readmission in this study (p = 0.37), the trend toward higher readmission in non-adherent patients may still be clinically meaningful. Medication non-adherence was a known risk factor for relapse in schizophrenia, and our results could have been limited by the retrospective design and relatively small sample size.

Likewise, while long-acting injectable antipsychotics were not significantly associated with reduced readmission risk (p=0.97), they are widely used in clinical settings to enhance treatment adherence. The absence of a significant association in findings may reflect real-world variability or documentation limitations rather than lack of clinical benefit. Future studies should explore these associations further in prospective larger scale cohorts.

While our findings compare some similarities with previous studies, certain differences. Warrant further exploration. For instance, we couldn’t find a statistically significant association between demographic variables such as age and gender with readmission, whereas earlier studies have reported these as important predictors. This discrepancy may be attributed to several factors. First, our relatively limited sample size may have lessens the statistical impact to identify such associations. Second, the homogeneity of our population in terms of socio-demographic characteristics could have reduced variability in those variables. Third differences in healthcare infrastructure and mental health service delivery in Saudi Arabia compared to western contexts may also contribute. For example, follow-up care practices, accessibility of community-based mental health services, and sociocultural perceptions of psychiatric illness may influence patient outcomes differently. These contextual differences might explain the variation in findings and highlight the importance of studying readmission predictors within specific local healthcare systems.

Additionally, the findings underscored no statistically significant correlations between the use of long-acting injectable antipsychotics and readmission rates within the current study. These results align with previous research by Kane et al. (43), which also indicated no significant statistical relationship, noting that while long-acting injectable antipsychotics correlated with reduced readmissions compared to oral alternatives, this may stem from limited sample sizes in both this study and Kane’s investigation. Effective injection antipsychotics have been linked to treatment compliance and have been shown in the literature to be predictive of psychiatric readmission (43).

There was likewise no significant correlation observed between the administration of electroconvulsive therapy at the time of discharge and 1-year readmission rates in this study. Nevertheless, existing research has highlighted that electroconvulsive therapy during the initial admission can serve as a predictor of subsequent readmission status.

Regarding discharge type, no associations were found linking the 1-year readmission rate at the point of first discharge in this study. Previous research has indicated that discharge planning and whether a discharge occurs against medical advice can serve as risk factors predictive of readmission (44). Additionally, the current findings exhibited no significant correlation between the duration of illness and readmission occurrences in the study population, contrasting observations made by Spring et al. that noted the duration as a contributing factor to readmission risk.

Deterioration in the ability and severity of psychiatric symptoms to perform social and occupational functions have been noted in studies such as that conducted by Stubbs et al. (45). However, assessing these functional abilities remained challenging within the parameters of the current study.

Existing literature indicates a range of readmission rates among patients diagnosed with schizophrenia, encompassing 33.3% to 86% after one to two years of follow-up. In the present investigation, the readmission rate within 1-year among patients with schizophrenia was identified at 36.6%, positioning it within the spectrum of previously reported rates.

Finally, addressing the second research question of this study, the findings from the multiple logistic regression analysis did not reject the null hypothesis, thereby failing to substantiate the alternative hypothesis. This outcome contrasts with previous studies (46, 47). The systematic review conducted by Sfetcu et al. categorized this risk factor as a predictor of psychiatric readmission. Additionally, the findings were consistent with Nelson et al., who associated outpatient care follow-up within 1-year with a significant impact on the 365-day readmission rate (48).

Even though the bivariate analysis showed a strong correlation between length of stay and readmission, this association did not persist in the multivariate logistic regression model. This attenuation may be explained by the inclusion of outpatient follow-up status, which showed a strong and independent association with readmission. It is possible that patients with longer hospital stays were also less likely to adhere to outpatient follow-up reducing the unique predictive value of hospitalization duration when both variables were considered together. This suggests that the observed relationship between length of stay and readmission in unadjusted analyses may have been confounded by follow-up care patterns.

The significant association between lack of outpatient follow-up and higher readmission risk may be explained by several factors. Patients who do not attend follow-up appointments may miss important opportunities for medication adjustments, early identification of relapse, and psychosocial support. Additionally, in the Saudi Arabian context, limited availability of community-based mental health services and potential stigma associated with mental illness could further discourage regular follow-up. These factors may increase the risk of deterioration in mental health status, ultimately leading to rehospitalization.

### 4.1 Study strengths

The findings of this study offer significant new knowledge in several areas. Firstly, it contributes to the understanding of the correlation between readmission rates and length of stay among patients with schizophrenia in psychiatric inpatient units located in Jeddah, Kingdom of Saudi Arabia. These results also lay the groundwork for mental health and psychiatric services in Saudi Arabia, directing future studies educational initiatives, outpatient follow-up and inpatient psychiatric care procedures. Ultimately, these results could enhance mental health care by improving the quality of life and accessibility for those with schizophrenia.

### 4.2 Study limitations

The current investigation has certain limitations. It made use of single-center retrospective data, which might have introduced selection bias and restricted how broadly the results could be applied. Additionally, admissions to other healthcare facilities and outpatient follow-up care were not evaluated. Future prospective, multi-center studies would provide a more complete picture of patient care and readmission risks.

The sample size was limited, and no priori sample size was calculation was done because of the unavailability of diagnostic prevalence data. This might be lowering the statistical power of the research and contributed to the absence of significant associations for several variables. Hence, non-significant findings should be interpreted with caution. To improve statistical validity and generalizability in further investigation, it is recommended to conduct *a priori* power analyses and recruit large samples, preferably through multi-center studies.

The study sample was predominantly male (84.8%0, which may reflect institutional admission patterns or gender related differences in treatment seeking behavior and hospitalization rates in the Saudi context. Although previous research has shown a higher prevalence of mental morbidities among females, the gender imbalance in our sample likely reflects inpatient admission trends rather than population level prevalence. This limitation may affect the generalizability of our finding to female patients.

Although outpatient follow-up was significantly associated with readmission (AOR: 2.266;95% CI;1.047-4.903) the wide confidence interval suggests imprecision in the estimate. This can be because of uneven distribution and limited sample size of follow-up status among participants and should be interpreted with caution.

### 4.3 Conclusions

This study investigated predictors of one-year psychiatric readmission among patients diagnosed with schizophrenia within a Saudi Arabian healthcare setting. The overall readmission rate was 36.6%. The most significant predictors identified were a higher number of previous hospitalization and lack of outpatient follow-up care. The length of inpatient stay and readmission however may be related according to the bivariate study. These findings emphasize the importance of systematic discharge planning and coordinated outpatient follow-up. Post-discharge treatment continuity can be strengthened by policy-driven approaches, such as requiring follow-up appointments before discharge, investigating community mental health outreach programs, and implementing digital remaining systems to improve appointment adherence. Understanding these risk variables can help enhance national mental health strategies and direct targeted interventions for high-risk individuals.

### 4.4 Recommendations

The results of the research shows that the particular actions are needed to reduce psychiatric readmissions among patients with schizophrenia, one such measure is to improve outpatient follow up care which can be accomplished by implementing remainder systems using SMS or phone calls, setting up follow-up appointments prior to hospital discharge and increasing access to community based mental health services, such as outreach programs or mobile clinics. For patient who struggle with mobility or transportation, telepsychiatry integration may improve continuity of care. Appropriate staffing inpatient and outpatient service coordination and digital infrastructure would be necessary for the implementation of these interventions. Additionally common barriers include treatment noncompliance, stigma around mental health and limited service accessibility should be addressed through culturally appropriate education and engagement strategies. The viability and efficacy of these interventions in the Saudi healthcare system and other comparable contexts should be investigated in future studies.

### 4.5 Future research

To extend the findings of the current study and achieve generalized results pertinent to this population, future research should encompass mental health facilities and psychiatric hospitals throughout Saudi Arabia. This includes key mental health institutions situated in various regions of the Kingdom. A coordinated effort in research development and service improvement is necessary to achieve common objectives in inpatient and outpatient services.

In addition to the aforementioned recommendations, future researchers should employ longitudinal and prospective designs aimed at identifying the risk factors contributing to relapse and readmissions. Utilizing cohort designs permits monitoring of patients over extended periods while making it possible to observe changes in readmission related to specific exposure variables over time, thereby minimizing recall bias. This approach will also enable clinicians to assess the readmission rate according to geographic location, healthcare setting, and distinct populations.

## Data Availability

The raw data supporting the conclusions of this article will be made available by the authors, without undue reservation.
